# The utility of goal attainment scaling in evaluating a structured water dance intervention for adults with profound intellectual and multiple disabilities

**DOI:** 10.1016/j.heliyon.2021.e07902

**Published:** 2021-08-30

**Authors:** Marie Matérne, André Frank, Patrik Arvidsson

**Affiliations:** a University Health Care Research Center, Faculty of Medicine and Health, Örebro University, SE 70185 Örebro, Sweden; b School of Law, Psychology and Social Work, Örebro University, Sweden; c Center for Adult Habilitation, Region Örebro County, Örebro, Sweden; d Region Gävleborg, Centre for Research & Development, Region Gävleborg, Sweden; e Centre for Augmentative and Alternative Communication, University of Pretoria, Pretoria, South Africa; f CHILD, Swedish Institute for Disability Research, Jönköping University, Sweden

**Keywords:** Profound intellectual and multiple disabilities (PIMD), Goal attainment scale (GAS), Intervention, ICF

## Abstract

**Background:**

Adults with profound intellectual and multiple disabilities (PIMD) have problems to be actively involved in essential life activities that affect their health. The aim of this study was to explore the utility of goal attainment scaling (GAS) in evaluating an intervention for adults with PIMD, and to describe how the GAS goals were set according to the International Classification of Functioning, Disability and Health (ICF) domains of body function as well as activity and participation.

**Method:**

As part of an aquatic intervention (Structured water dance), 28 adults with PIMD received GAS goals which were adapted to their individual needs and which the intervention could affect.

**Result:**

Twenty of the goals were formulated within the ICF Activity/Participation domain and eight within the Body Functions domains. On average, participants improved by 1.25 levels on the five-level GAS scales.

**Conclusion:**

GAS can be a useful tool for setting and evaluating individualized and meaningful goals, in body functions as well as in activity and participation, related to a healthpromoting activity for adults with PIMD.

**What this paper adds**

• The findings of this paper contribute to the knowledge of the utility of Goal attainment scaling (GAS) for adults with profound intellectual and multiple disabilities (PIMD).
• When setting goals adults with PIMD need assistance, due to their disability, from a support person or a disability health care professional.
• Even small changes are probably meaningful to consider for this group and could be measured by GAS.
• Goals according to GAS are useful in the evaluation of an aquatic activity
• GAS goals were mostly formulated within the activity/participation domain of ICF.

## 1 Introduction

People with profound intellectual and multiple disabilities (PIMD) have a combination of intellectual disability and lifelong physical impairments (Granlund et al., 2014; Nakken and Vlaskamp, 2007; World Health Organization, 2001). In addition, they have other medical problems (Van Timmeren et al., 2017) such as epilepsy and spasticity, as well as visual, auditory and communicative impairments that together cause severe health issues and fundamental problems in many areas of life (Granlund et al., 2014; World Health Organization, 2001). Due to the combination of their disabilities, and their health issues, people with PIMD have activity and participation limitations (Hanzen et al., 2017; Hanzen et al., 2018). Therefore it is important to develop new interventions for the prevention of further health decline in this group (World Health Organization, 2001).

When adults with PIMD wish to be actively involved in everyday activities they are, due to their severe impairments, always dependent on direct support from professionals (Haines et al., 2018; Hanzen et al., 2017, 2018, 2020). However, through individualized training and adaptations as well as through e.g. interventions focusing on attitudes among the professionals, individuals with PIMD can participate and be actively involved in essential aspects of life (Arvidsson et al., 2014; Hanzen et al., 2017, 2018). Thus, both the domain of body functions (also possible impairment) and the domain of activity and participation (also possible activity limitations and participation restrictions) in the International Classification of Functioning, Disability and Health (ICF) are highly relevant for intervention goals (Granlund et al., 2014; World Health Organization, 2001). According to the ICF, participation as an essential aspect of health, can be operationalized as actual performance in everyday activities and can also be assumed to be a reflector of the interaction between an individual's functions (and impairments) on one hand and the individualized support (being available or not) on the other (Granlund et al., 2021; World Health Organization, 2001). In order to make it even more relevant for adults with PIMD, an operationalisation of participation should thus not only include actual performance in existing activities but also include possible involvement in new daily activities (Hanzen et al., 2017; World Health Organization, 2001). However, due to communicative and cognitive limitations in for example imagining possible new activities to be involved in, adults with PMID will need support to be able to both set and evaluate relevant goals (Granlund et al., 2014; Hanzen et al., 2018, 2020; Talman et al., 2021).

According to the national guidelines for disease prevention methods issued by the Swedish National Board of Health and Welfare (Socialstyrelsen, 2011), activities for adults with PIMD, just as for the general population, should be based on health promotion and disease prevention. Reasons for emphasizing prevention are to reduce secondary problems, provide leisure activities and improve quality of life (Rimmer, 1999).

Hydrotherapy, or other aquatic exercises in a warm pool, preferably at a rehabilitation centre or hospital, is one frequently requested activity for adults with PIMD (Mulligan and Polkinghorne, 2013). The structured water dance intervention (SWAN) is a new group activity adapted to adults with PIMD (Lundqvist et al., 2020) and is influenced by hydrotherapy and floor dance (Hagström et al., 2011). The SWAN intervention evaluates the effect on outcomes such as stress, wellbeing, hypertonia, pain, and social interaction in this target group.

The usage of goal attainment scaling (GAS) is well established in the field of rehabilitation, and the method is frequently used in Sweden (Kiresuk et al., 1994; Socialstyrelsen, 2012). GAS evaluates the degree to which a person achieves identified individual goals through an intervention (Kiresuk et al., 2014; Kiresuk et al., 1994). GAS facilitates individual goal setting and is considered especially sensitive in capturing individual change over time (Gordon et al., 1999; McLaren and Rodger, 2003; Mithcell and Cusick, 1998; Russell et al., 1995).

Despite the common use of GAS in clinical practice and in research, confidence in the results of studies using this method can be low because of the great variability in how researchers apply it (Krasny-Pacini et al., 2016). Furthermore, there is a lack of studies attempting to use GAS to facilitate individual goal setting and to describe individual goals for adults with PIMD in relation to participation in existing or new activities in everyday life (Dijkhuizen et al., 2019; Jones et al., 2006; Nguyen et al., 2019; World Health Organization, 2001). Jones et al. conducted a study including persons with PIMD where GAS goals were set in areas of potential improvement including access to community-based experiences, but most goals were related to behaviour, health and physical competence. Thus it is interesting to understand how the goals set within GAS are distributed from an interactional perspective described on the basis of the model of the ICF, it the goals can be classified as goals in terms of body functions (or impairment) or activity and participation (activity limitations and participation restrictions) (World Health Organization, 2001).

The first aim of this study was to explore the utility of GAS as a tool for facilitating individual goal setting and in the evaluation of individual goal attainment in adults with PIMD participating in the SWAN. The second aim was to describe how the GAS goals were set according to the ICF domains of body functions as well as activity and participation.

## 2 Method

### 2.1 Study design

This study is part of a larger research project of an aquatic intervention, SWAN (Lundqvist et al., 2020), which is a randomised multicentre study with a crossover design. GAS was applied as a measure of individual goal attainment along with other measurements in the evaluation of the proposed effects of SWAN. The present study focuses on the utility of GAS for the participants in the SWAN intervention. The GAS goals were set before the start of the intervention period for each participant and evaluated mid-session and after the final session. The participants received one GAS goal each.

### 2.2 Participants

The participants were recruited from adult habilitation centres in four Swedish regions by licensed physiotherapists working for the SWAN research project. Originally, there were 34 individuals participating in the SWAN, nine participants from each of two centres and eight participants from each of the other two centres. The intervention involved eight SWAN groups with four to five participants in each group.

The inclusion criteria for this study were being 18 years or older, having PIMD (according to (Nakken and Vlaskamp, 2007)) and having previous experience of interventions in warm water with no discomfort related to such activities (Lundqvist et al., 2020). The exclusion criteria were having severe hearing impairment (since hearing the music is a prerequisite for taking part in the intervention) and having infections or ulcers that would be infectious in the pool.

The mean age was 33.5 years and ranged from 21 to 53. Apart from profound intellectual disability as an overall diagnosis, cerebral palsy was the most common specific diagnosis associated with PIMD (18 of 28 participants). Other diagnoses were epilepsy, hydrocephalus, cerebrovascular disease, autism and corpus callosum agenesis. The information about diagnosis was reported with the help of a support person (member of the residential care staff or personal assistant) or a family member, who also filled in a form with demographic data. Where information was lacking or not clear, the physiotherapist in the research team asked the local physiotherapists or SWAN leaders to look up the medical records for complementary data.

### 2.3 Structured water dance (SWAN)

The SWAN (Lundqvist et al., 2020; Materne et al., 2021) was developed by combining the advantages of hydrotherapy (Geytenbeek, 2002) and floor dance (Hagström et al., 2011). SWAN is performed as a group activity for four to five adults with PIMD in a warm pool, led by two SWAN instructors. Each participant has one support person in the water acting as a dance partner and one at the poolside. All the participants with PIMD use floating devices during the water dance session for safety and stability. One SWAN session includes a variety of dance themes accompanied by a playlist of nine songs. The dance movements are designed to be performed by the support person (dance partner) moving the participant's body due to the severity of the participant's impairments. The SWAN intervention consists of 12 sessions, one time per week.

### 2.4 Goal attainment scaling (GAS)

GAS (Kiresuk et al., 1994, 2014) measures the degree of attainment of individualized goals, where the 0 level represents the expected outcome, +2 represents “much better than expected and -2 represents “much worse than expected”. The -2 level maybe considered as equal to baseline. Using the SMART (Bovend'Eerdt, Botell and Wade, 2009) approach is recommended in order to make the goal specific, measurable, achievable, realistic/relevant and timed. Goals should also be formulated in collaboration with the person being subject to the GAS method.

### 2.5 Procedure and data collection

Before starting the SWAN, the physiotherapist (intervention leader) from the research team (AF) together with another member of the research team met the participant and his or her parent, legal guardian, member of the residential care staff or personal assistant. In most cases, the local physiotherapist was present. The meeting took place at the included habilitation center, where one participant at a time was scheduled to discuss and identify desirable and expected individual goals. However, the actual goals were not set at that point. The support persons were not familiar with GAS and the local SWAN instructors did not have any specific education in setting goals according to GAS. The focus of the discussion was to identify, with help from the support persons, a situation in everyday life that could be affected by SWAN and to establish the baseline level for the individualized GAS in order to evaluate the study participant's individual goal achievement. One goal was suggested for each participant. The intervention leader (AF) and the co-researcher (MM) later discussed the proposed goals further at the research centre, because of time constraint, and in most cases they would together formulate the goals at the different levels, from -2 to +2. In some cases, the researchers discussed the goal by phone with a support person or a parent to confirm they had understood the situation, clarifying the goal. After SWAN session 6, the mid-intervention GAS assessment was performed. The intervention leader (AF) informed all participants and their support persons about the assessment and handed out a copy of the individual goal description to each participant which was the first time they received the scale in written form. The support persons were asked to help the participants to grade their achievement of the individual goal by sending a text message to the physiotherapist (AF) within the days following the SWAN session 6. The final evaluation was completed in the same way after session 12 of the SWAN and thus no further evaluation was conducted.

GAS goals were revised in two cases when performing the mid-intervention GAS assessment because the researchers had misinterpreted the intention of the goal. In the result section, we have provided examples that showed improvement, where scales had five score levels, and where the participant attended more than half of the sessions.

### 2.6 ICF

A second aim of this study was to describe how the goals were set according to the ICF (World Health Organization, 2001). The ICF includes a model for describing and organizing information on functioning and disability for practitioner and researchers. It takes into account the role of environmental factors in disability, as well as health conditions and their effects on the individual. The authors classified all the goals from an ICF perspective by linking the described goals to a specific ICF code (Cieza et al., 2019; Cieza et al., 2005).

### 2.7 Ethical considerations

The regional Ethical Review Board in Uppsala, Sweden (ref. 2018/070) approved the study. All the participants' legal guardians (a parent or other appointed person) gave written consent before starting the intervention, since adults with PIMD included in this study do not have the intellectual capability to independently give their consent about participation. The participants and their legal guardians were informed of the study and the possibility to withdraw their participation, in accordance with the Declaration of Helsinki (World Medical Association, 2017).

## 3 Results

### 3.1 Attendance

From the beginning, it was 34 participants; four participants did not meet our inclusion criteria of having PIMD, because their motor functioning was not severely limited, and one individual did not meet the inclusion criterion of having profound intellectual disability. One participant did not have result of the GAS scoring from session 12 and was excluded from this study. Thus, 28 adults (10 women and 18 men) with PIMD were included in this study. On average, the participants attended 9.5 of the 12 sessions (ranging from 5 to 12) in the SWAN. During the first six sessions (sessions 1–6) the mean attendance was 4.8 (range 2–6) and during the last six sessions (sessions 7–12) the mean attendance was 4.7 (range 0–6).

### 3.2 Description of individual goal setting

In Table 1, all 28 individualized GAS scales (one per participant) are presented at the baseline level (-2 on the scale), and linked to the ICF code and classified to the ICF domain.

**Table 1:** Baseline description and ICF classifications for each participant's GAS scale.

ICF component and domain (number of goals)	Baseline description and ICF classification
Health condition (n = 1)	1) Has 20–30 epileptic seizures per day. (b110)
BODY FUNCTIONS (n = 7)	2) Has spastic seizures at least 5 times per day. (b780) 3) Sleeps poorly at night. He is tired in daytime, even during activities and often falls asleep when there is no ongoing activity. (b134) 4) Sleeps poorly at night. Her alertness is decreased in daytime and she falls asleep in her wheelchair at least once per day, even during activity. (b110 + b134) 5) Often tired in daytime. Falls asleep even during ongoing activity. Very uneven sleep during the night. (b134) 6) Difficulty falling asleep. Often wakes up at night. Very tired in daytime. (b110 + b134) 7) Sleeps poorly. Sometimes no sleep at all. Takes melatonin. (b134) 8) Often tired and shows interest only when someone is addressing him directly. (b110 + b134)
ACTIVITY AND PARTICIPATION	
General tasks and demands (d210-d299) (n = 3)	9) Always stressed and screams many times during pool activity. (d240) 10) Often stressed during the morning routine and often has difficulty cooperating. (d230) 11) Always sensitive to stress (d240) and always has difficulty relaxing. (b765)
Mobility (d410-d499 (n = 11)	12) Often misaligned in her wheelchair and needs adjusting 5–7 times per day. (d415) 13) Tense, with involuntary movements. The assistants need to help him adjust his seating position in the wheelchair 5–10 times per hour. (d410) 14) Due to spasticity, he has difficulty in keeping his seating position. The staff have to alter his position at least once per hour. (d415) Often complains about pain, scoring between 5 and 10 on a 1–10 visual-analogue scale. (b280) 15) His left arm is most of the time pulled up against his chest with elbow flexed. He can let his arm down by himself sometimes or when reminded. (d410 or d429) 16) Can sometimes stand up by himself, e.g. from the bed. (d410) He almost always needs assistance when he cannot rely on his legs because of epilepsy or fatigue. (d415) He always wakes up at night. (b134) 17) It is difficult for staff to position her on her side in her bed. She resists and gets upset almost every time. (d 415) 18) Has increased muscle tone, making it difficult to turn him over on his side in his bed. (d410). He needs a lot of support with pillows to keep him positioned. (d415) 19) Very tense when turning in bed. Needs help from two assistants to support his head and body. He shows sign of discomfort. (d410) 20) Sits with arms flexed almost all the time. They are only at rest when he gets tired and falls asleep. (d415) 21) Spastic in the extensor muscles of the legs corresponding to Ashworth 3. (b) Slides forward in the wheelchair. Needs help to adjust her seating position several times during the day. (d415) 22) Spastic in the extensor muscles of the legs corresponding to Ashworth 4 and high muscle tone in the neck (b735). Needs help occasionally to adjust her seating position during the day. (d415)
Self-care (d510-d599) (n = 6)	23) Has very limited mobility and resists the staff while they are dressing him. He can sometimes take an active part when urged. (d540) 24) It is difficult dressing him. It takes a long time and he needs help from two assistants. (d540) 25) When he is about to dress he stands while his assistant supports him. He crouches while the assistant is pulling up his trousers. (d540) The assistant is unsure if he is going to fall. He is able to stand for a maximum of 30 s. (d415) 26) Dressing is difficult. (d540) He becomes very tense in his arms (Ashworth 3) (b735) 27) Gets more spastic in the flexor muscles of her arms during dressing. It is difficult for staff to help her. She looks bothered. (d540) 28) Very tense when showering. The assistants have difficulty washing him everywhere. (d510)

(GAS, goal attainment scaling; ICF, International Classification of Functioning, Disability and Health.).

Twenty-one of the participants had a GAS goal setting on a five-level scale from -2 to +2 (Bovend'Eerdt et al., 2009; Russell et al., 1995); the remaining seven had a goal setting with only 3 levels (-2, 0 and +2) of the GAS scale because in these cases the goal wasn't amenable to formulating and predicting five attainment levels (Krasny-Pacini et al., 2017).

### 3.3 Goal attainment

**Figure 1:**
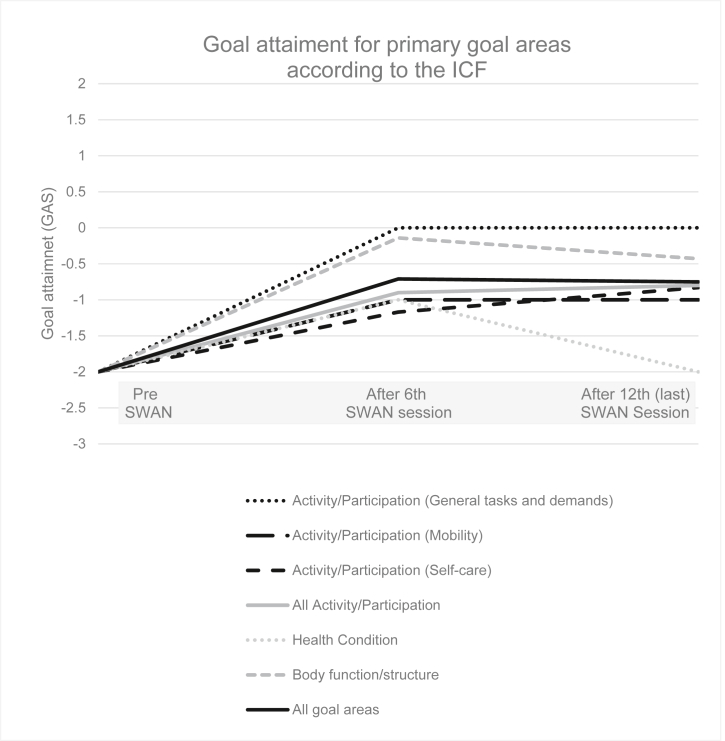
Goal attainment level pre-SWAN and after the sixth and 12th SWAN session.

The participants' GAS levels were followed up twice during the intervention period (after sessions 6 and 12). Figure 1 and Table 2 show all participants' GAS levels before the intervention, midway (after session 6) and at the final evaluation (after session 12). The mean increase after the 12-week intervention period was +1.25 across all goal areas (from -2 at baseline to -0.75 after session 12). After session 6, the mean increase was +1.29 (from -2 to -0.71) and the change in the GAS scores then levelled out. The highest increase was shown in the activity/participation domain *General tasks and demands*, showing a mean increase of +2.0 (from -2 to 0). Three participants had a goal within this domain, which was related to levels of stress.

**Table 2:** GAS level pre-SWAN, after the sixth and 12th SWAN session.

Goal area	Primary goal area according to ICF domain	n	GAS level		
			Pre-SWAN	After session 6	After session 12
Goals related to ICF Activity and Participation component	General tasks and demands	3	-2	0	0
	Mobility	11	-2	-1	-1.0
	Self-care	6	-2	-1.17	-0.83
	*All goals related to Activity and Participation*	*20*	*-2*	*-0.9*	*-0.80*
Goals related to ICFs Body Functions and Body Structures component	Health Condition	1	-2	-1.0	-2.0
	Body Functions and Body Structures	7	-2	-0.14	-0.43
	*All goals related to health condition and Body Functions and Body Structures*	*8*	*-2*	*-0.25*	*-0.63*
All goal areas		*28*	*-2*	*-0.71*	*-0.75*

The italic values are represents the (mean value).

Figure 1 shows that although goal attainment scores levelled out in most goal areas after session 6, in the area of self-care activities they carried on improving beyond session 6.

The primary goal areas were described according to ICF component and domain. (GAS, goal attainment scaling; ICF, International Classification of Functioning, Disability and Health).

At the midway evaluation, 12 participants (43%) had increased at least two goal attainment scale levels (to 0 or higher) and after the final session (session 12), 11 participants (39%) had increased at least two scale levels from baseline. Moreover, 17 participants (61%) had increased at least one scale level (to -1 or higher) after session 6 and 15 participants (53%) had increased at least one scale level after the final session.

### 3.4 Description of the individual attainment of goals

In Tables 3, 4, and 5, GAS scales representing the different ICF components and domains are presented. We have chosen examples where improvement was attained, where the GAS scale had five scoring levels and where the participant attended more than half of the sessions. The achieved level of goal attainment after session 12 is highlighted in bold in each table.

**Table 3:** GAS outcome: ICF domain Sleep (Body Functions component).

Goal attainment level	Individual goal level descriptions: goal 3
Much better than expected outcome +2	Sleep is satisfying. He is awake during activity and awake when there is no ongoing activity (except for rest periods).
Somewhat better than expected outcome +1	**Sleeps better. He is less tired in daytime, awake during activity and less tired when no activity is ongoing.**
Expected outcome 0	Sleeps better. He is tired in daytime and often falls asleep when there is no ongoing activity but stays awake during activity.
Somewhat worse than expected outcome -1	Sleeps better. He is tired in daytime, even during activities and often falls asleep when no activity is ongoing.
Much worse than expected outcome (baseline level) -2	Sleeps poorly at night. He is tired in daytime, even during activities and often falls asleep when no activity is ongoing.

**Table 4:** GAS outcome: ICF domain General tasks and demands (Activity and Participation component).

Goal attainment levels	Individual goal level descriptions: goal 10
Much better than expected outcome +2	Seldom stressed during the morning routines and seldom has difficulty cooperating
Somewhat better than expected outcome +1	Sometimes stressed during the morning routines and seldom has difficulty cooperating
Expected outcome 0	**Sometimes stressed during the morning routines and sometimes has difficulty cooperating**
Somewhat worse than expected outcome -1	Often stressed during the morning routines and sometimes has difficulty cooperating.
Much worse than expected outcome (baseline level) -2	Often stressed during the morning routines and often has difficulty cooperating

**Table 5:** GAS outcome: ICF domain Mobility (Activity and Participation component).

Goal attainment levels	Individual goal level descriptions: goal 19
Much better than expected outcome +2	Less tense when turning in bed. Needs help from one assistant. Shows no sign of discomfort.
Somewhat better than expected outcome +1	**Less tense when turning in bed. Needs help from two assistants. Shows no sign of discomfort.**
Expected outcome 0	Very tense when turning in bed. Needs help from two assistants but they do not need to support her head. Shows no sign of discomfort.
Somewhat worse than expected outcome -1	Very tense when turning in bed. Needs help from two assistants to support her body and head. Shows no sign of discomfort.
Much worse than expected outcome (baseline level) -2	Very tense when turning in bed. Needs help from two assistants to support her body and head. Shows signs of discomfort.

Table 3 describes the GAS outcome for a 50-year-old man with cerebral palsy. He had trouble sleeping at night. This poor sleep made him tired even in daytime. The expectation was that water dance, with its components of physical activity, could perhaps improve his sleep.

There could be many factors in this man's life affecting the results. However, after 12 SWAN sessions, his sleep was better and he was less tired during daytime.

Table 4 describes the GAS outcome for a 27-year-old man with cerebral palsy. Before the SWAN intervention, he was stressed during the morning routines and it was hard for him to cooperate. During the discussion of goal setting we agreed that water dance with different movements in warm water might help him to relax.

The result showed that the stress and cooperation difficulties during the morning routines had changed for the better after the SWAN intervention.

Table 5 shows the GAS outcome for a 26-year-old woman with cerebral palsy. She was very tense and disturbed when her two assistants turned her in bed while supporting her body and head.

After the final SWAN session, this woman was less tense when being turned in bed by her two assistants, and she showed no sign of discomfort.

## 4 Discussion

This paper describes the utility of GAS as a method for facilitating individual goal setting and for evaluating the attainment of individualized goals related to an aquatic intervention for adults with PIMD. The results show that, on average, the 28 participants improved their goal attainment by 1.25 levels. The goals were described according to a specific ICF code and domains in the different components of the ICF (Krasny-Pacini et al., 2016; Nguyen et al., 2019). *Mobility* is the ICF domain that represented most of the participants' goals, and this is not surprising because the intervention was primarily a physical activity. According to Bovend'Eerdt et al. (2009), it is easier to focus on goals relating to the ICF Activity and Participation component because they are often easy to measure, and other studies also confirm that goals relating to mobility and active movement are the most common when studying children with cerebral palsy (Bexelius et al., 2018; Krasny-Pacini et al., 2016). Similarly, in our study, 20 of 28 goals were within the ICF activity and participation component. Furthermore, we found that the three goals in the domain *General tasks and demands* showed the largest increase (mean 2.0). This is interesting considering that they all concerned stress, and the SWAN intervention actually addressed outcomes such as stress and wellbeing. The goals set in the domain *Self-care* did not level out after the sixth session, but rather increased. This may indicate that it was a prioritized area which the assistants and participants were highly motivated to improve, and which furthermore could improve both the assistants' work situation and the daily life of the person with PIMD.

Due to communicative and cognitive limitations, setting and evaluating goals, especially in a not well-known activity, is challenging for people with PIMD and they need support to be able to do that (Granlund et al., 2014; Hanzen et al., 2017, 2018, 2020). For adults with PIMD, however, participation in everyday life is most often achieved through necessary support from others, for example staff members, personal assistants, or family members (Hanzen et al., 2017, 2018; Johnson et al., 2012). This reliance of supportive persons could of course imply that the chosen goal is not the participant's own first choice. However, in our study, there was always a support person close to the participant, who discussed appropriate goal areas with the intervention leader and a researcher from the project, who could adjust the GAS goal if needed. The exact wording and scoring of the GAS goal was not communicated with the legal guardian, the support person and the participant before the intervention started. This could be a possible limitation that could lead to a less focused and accurate goal attainment.

The improvement of GAS scores by 1.25 levels in the current study has to be interpreted with caution, but it indicates that GAS can be used in the evaluation of rehabilitation and habilitation interventions for people with PIMD. Steenbeek and colleagues (2005) concluded in their study about children with cerebral palsy and treatment with botulinum toxin that GAS should improve at least two levels in median during an intervention of 10 weeks to make a clinically relevant difference. The target group in our study has limited access to individualized rehabilitation and fewer opportunities to take part in habilitation activities (Hayton and Dimitriou, 2019), and one reason for this may be that people with PIMD are underestimated both as goal setters as well as evaluators of their goal achievement.

The validity of GAS is dependent on the therapist's experience, objectivity and ability to anticipate the possible outcomes based on his or her knowledge of the patient (McLaren and Rodger, 2003), and consequently, questions about validity should be raised for every study using GAS (Krasny-Pacini et al., 2016). There is a risk of bias when conducting research using GAS and when constructing goals in several steps. The different levels may be formulated so that higher levels are too easy to reach or the goal may be formulated in imprecise terms (“better than”, “worse than”) which may bias the scoring to a more favourable attainment level (Krasny-Pacini et al., 2016). In our case, AF is an experienced physiotherapist who has worked with the target group for over 25 years. However, most goals were levelled out after the mid-intervention evaluation, which could mean that we could have set new goals for the period after the mid-intervention and involve the participants more in the decision making processes (Talman et al., 2021).

Moreover, King et al. (2000) proposes strategies to address the issue of validity: to use other standardized measurements alongside GAS and to use randomly selected control goals. In the larger SWAN project we used complementary measurements, both standardized assessments for collecting objective data, and questionnaires for collecting subjective data (Lundqvist et al., 2020). Including the target person, with assistance of the support person, in setting the goals according to the GAS method is also considered to strengthen validity by making the goals meaningful and relevant (Kiresuk et al., 2014). Training of raters is another strategy that will improve validity and reliability (Kiresuk et al., 2014; Krasny-Pacini et al., 2016). In our case, two of the researchers (AF and MM) had training in formulating GAS goals. Even though the support persons were not familiar with GAS and even though the local SWAN instructors did not receive any specific education in setting goals according to GAS, the structure of GAS seemed to be useful in the goal-setting process. However, to determine to what amount the GAS as such facilitated the goal setting, or if it e.g. were the discussion in connection to the goal setting that the physiotherapist (AF) had with the support persons, need to be further examined, suggestable in a controlled study.

In a study about brain injury rehabilitation it was found that technical errors during the construction of GAS goals could reduce the validity of GAS as an outcome measure (Grant and Ponsford, 2014). To minimize these errors, a checklist could be provided as well as training. A checklist for judging the quality of GAS has been developed by Krasny-Pacini et al. (2016), who proposed a set of 17 criteria. These criteria are intended for research rather than clinical contexts, addressing validity and reliability criteria, other factors such as the Specific, Measurable, Achievable, Relevant and Timed (SMART) criteria, and the ICF categorization of goal types (Talman et al., 2021; World Health Organization, 2001). Using the ICF to classify the participants' goals can be considered a strength because it directly relates the goal to the person's functioning and health.

A Swedish study on the quality of goal setting using GAS found that the quality of goals from disabled children in the clinical practice was relatively high (Bexelius et al., 2018). This study also suggested using the SMART criteria to increase the quality of goal setting (Bexelius et al., 2018), which has also been encouraged by other authors (Bovend'Eerdt et al., 2009). The present study was influenced by the SMART approach but did not systematically follow the criteria.

All GAS scales were constructed using -2 as the baseline, but within a group of people with profound disabilities, it is reasonable to think that no improvement or even a deterioration can occur and thus -1 could be used as the baseline (current level); then -2 could be defined as worse than the current level (Bovend'Eerdt et al., 2009; Jones et al., 2006). This may be even more relevant for degenerative disorders. One criticism of GAS is that the five-level scale is difficult and time-consuming to construct (Grant and Ponsford, 2014). One study examined the use of a three-level scale in scoring the attainment of individual goals (Krasny-Pacini et al., 2017). They found the method feasible within a clinical setting and that it was possible for therapists to predict treatment outcomes. Our experience of using GAS in the present study was also that a three-level scoring scale was sufficient in seven cases.

### 4.1 Study limitations

∗ The study sample was restricted to 28 participants who could not communicate verbally and therefore were dependent on their family or support persons to interpret their needs in constructing the individual goals.
∗ The intervention leader (a physiotherapist) who constructed the GAS goals did not have the same knowledge about the participants as the local physiotherapists.
∗ The intervention may be assumed to primarily focus on motor function and physical activity by the support persons because of the setting in a rehabilitation pool and because the intervention leader and most of the instructors were physiotherapists. This may have affected the results, since nearly half of all goals were categorized within the motor function area.

## 5 Conclusion

GAS has the potential to be utilized as a method for evaluating individualized goals for people with PIMD in relation to an intervention aiming to improve various health aspects. Twenty of the goals in this study were formulated within the ICF activity and participation domains and eight within the body functions domains. This diversity of goals across ICF domains indicates that there was room for individual adaptations of the scales to the adult's needs and abilities. On average, participants improved by 1.25 levels on the GAS scales after a maximum of 12 sessions of the intervention. We can conclude that GAS seems to be useful in the evaluation of goal attainment in adults with PIMD who participated in the intervention. Even small changes could be measured with GAS that makes it a useful tool to evaluate different improvements for adults with PIMD in body functions as well as in the participation in essential everyday activities, but further research is needed.

## Declarations

### Author contribution statement

Marie Matérne, André Frank and Patrik Arvidsson, Conceived and designed the experiments; Performed the experiments; Analyzed and interpreted the data; Contributed reagents, materials, analysis tools or data; Wrote the paper.

### Funding statement

This work was supported by Regional Research Council in the Uppsala–Örebro Region and Region Örebro län.

### Data availability statement

Data will be made available on request.

### Declaration of interests statement

The authors declare no conflict of interest.

### Additional information

The clinical trial described in this paper was registered at ClinicalTrials.gov under the registration number NCT03908801.
